# Stroke onset time affected outcomes in the young acute ischemic stroke patients treated with endovascular thrombectomy

**DOI:** 10.3389/fneur.2025.1655646

**Published:** 2025-10-23

**Authors:** Mengying Yu, Jie Lin, Asta Debora, Kai Lin, Mengqi Lin, Ru Lin, Haoli Xu, Mo Zheng, Ying Zhou, Fei Yao, Kuikui Zheng, Yingbao Huang, Huwei Xia, Yunjun Yang, Nengzhi Xia

**Affiliations:** ^1^Department of Radiology, The First Affiliated Hospital of Wenzhou Medical University, Wenzhou, Zhejiang, China; ^2^Department of Nuclear Medicine, The First Affiliated Hospital of Wenzhou Medical University, Wenzhou, Zhejiang, China; ^3^Department of Radiology, The People's Hospital of Yuhuan, Yuhuan, Zhejiang, China; ^4^Key Laboratory of Novel Nuclide Technologies on Precision Diagnosis and Treatment & Clinical Transformation of Wenzhou City, Wenzhou, Zhejiang, China

**Keywords:** Ischemic stroke, endovascular thrombectomy, circadian rhythm, malignant brain edema, young age, outcome

## Abstract

**Objective:**

The purpose of this study was to investigate the association between stroke onset time and prognosis after endovascular thrombectomy (EVT) in Acute Ischemic Stroke (AIS) patients at different ages.

**Methods:**

The AIS patients who underwent endovascular thrombectomy (EVT) between August 2018 to June 2022 were collected retrospectively. The patients were divided into two onset time groups [day-onset (6:00 h−18:00 h) vs. night-onset (18:00 h−6:00 h)], and further divided into 4 onset age groups (< 55 y, 55–64 y, 65–74 y and 75+ y). The primary outcome was discharge National Institutes of Health Stroke Scale (NIHSS) score and secondary outcomes were malignant brain edema, hemorrhagic transformation (HT), and early vascular recanalization (mTICI ≥ 2b). Mediation analyses were applied to explore how malignant brain edema, HT, and early vascular recanalization affect the relationship between onset time and outcome.

**Results:**

A total of 470 AIS patients were enrolled, of whom 68 patients were younger than 55 years. After adjusting for confounders, younger (< 55 y) day-onset AIS patients had worse discharge outcomes (discharge NIHSS≥16, OR = 0.136, 95%CI = 0.027–0.678, *P* = 0.015) and were more prone to neurological deterioration (ΔNIHSS ≥ 4, OR = 0.081, 95%CI = 0.012–0.544, *P* = 0.010), malignant brain edema (OR = 0.145, 95%CI = 0.027–0.798, *P* = 0.026), and HT (OR = 0.231, 95%CI = 0.057–0.946, *P* = 0.042), while early vascular recanalization was less likely to occur (OR = 0.118, 95%CI = 0.017–0.813, *P* = 0.007). Mediation analysis showed that stroke onset time → malignant brain edema → discharge NIHSS score pathway was significant (c' = −2.029, BC 95%CI = −6.217; −0.087).

**Conclusions:**

The outcomes of night-onset young AIS patients treated with EVT were better than day-onset, which may be related to the diurnal difference of malignant brain edema. However, these results were related to a small subgroup of patients and given the methodological statistical limitations (multiple testing correction), these results are only driving hypothesis. Further research with larger patient sizes are required to validate these results and explore the underlying mechanisms.

## 1 Introduction

Circadian biology regulates almost every aspect of mammalian physiology, pathology, and the therapeutic efficacy of diseases. In stroke, circadian biology may impact disease susceptibility, injury degree, and therapy response (1). A recent study suggested that the challenges of translating neuroprotective treatments of stroke from the lab to clinical trials might be due to a mismatch between the circadian rhythms of animal models and patients in clinical trials (2). Compared to the failures of neuroprotective strategies, intravenous thrombolysis and endovascular thrombectomy (EVT) have been effective in clinical trials, suggesting that reperfusion is a valid treatment on both awake and non-awake strokes (2). Recently, EVT has been regarded as a standard therapy for vessel recanalization after stroke (3). However, clinical research on the correlation of stroke onset time and stroke outcome have been limited and inconsistent. Wang et al. (3) found that acute ischemic stroke (AIS) patients treated with EVT, whose onset time were between 00:00 h and 12:00 h, had a higher proportion of 3-months good outcomes. Ryu et al. (4) found that night-onset AIS patients presented higher neurological severity and worse outcomes. A recent study based on melatonin levels in stroke patients suggested that melatonin secretion levels affect stroke outcomes (5). Whereas Ding et al. (6) found that circadian rhythm had no association with the outcome of AIS patients treated with recombinant tissue plasminogen activator. Therefore, the relationship between stroke prognosis and circadian rhythm remains to be further investigated.

Stroke is considered a disease of the elderly in the prevailing view since published reports indicated that the majority of stroke cases occurred in the elderly (7). However, the global incidence of stroke is gradually becoming younger (8, 9), and the incidence of ischemic stroke in young patients has reached a significant increase in recent years. A previous England study compared people living in Oxford in 2002–2010 vs. 2010–2018 and found that there was a significant increase in stroke incidence among people under the age of 55 (10). The possible reason may be that lifestyle-related risk factors for stroke are more common in the younger population, such as excessive alcohol consumption, smoking, and illicit drug use (11). Stroke in the young imposes a greater financial burden on society than stroke in the elderly, as the disease cripples them during their most productive years. A previous study has shown that circadian rhythmic activities change markedly as advancing age (12), indicating that circadian rhythm is related to age. The amplitude of many rhythms weakened in the elderly relative to younger adults. A recent study published in *Science* examined the age and sex differences in the expression of genes related to circadian clocks. They found that younger people had stronger circadian rhythms than older people (13). Further studies on the relationship between age, circadian rhythm, and stroke prognosis are needed in order to provide better management for AIS patients.

Therefore, the purpose of this study was to investigate the association between stroke onset time and outcomes after EVT treatment in AIS patients at different ages. We hypothesized that circadian rhythm differences were more pronounced in younger stroke patients and were independently associated with outcomes.

## 2 Materials and methods

### 2.1 Patients selection

Data on patients with AIS admitted to The First Affiliated Hospital of Wenzhou Medical University from August 2018 to June 2022 were reviewed retrospectively. Inclusive criteria were as follow: (1) AIS of anterior or posterior circulatory large vessel occlusion confirmed by CTA or DSA; (2) No intracranial hemorrhage or cerebral edema observed on baseline CT; (3) Received EVT treatment within 24 h of onset; (4) Follow-up CT after surgery; (5) Complete laboratory and clinical data; (6) No deaths occurred during the hospitalization period. Thirty five patients were excluded for the following reasons: (1) Unrecorded operation time (*n* = 8); (2) Unknown medical history (*n* = 6); (3) Loss of baseline CT images (*n* = 6) and (4) Poor quality of CT images, causing difficulty to validate image characteristics such as brain edema and hemorrhagic transformation (*n* = 15). Finally, a total of 470 cases were enrolled. The patients were divided into two- groups [day-onset (6:00 h−18:00 h) vs. night-onset (18:00 h−6:00 h)] according to the onset time of stroke, or 4 age groups (< 55 y, 55–64 y, 65–74 y and 75+ y) based on onset age (9). Stroke onset time was defined as time of first observed neurological deficits by patients or their relatives. The flowchart of the study was showed on Supplementary Figure 1. Our study was approved by the Medical Ethics Committee of The First Affiliated Hospital of Wenzhou Medical University. The written informed consent was waived due to the retrospective design.

### 2.2 Data collection

We retrieved demographic and baseline clinical information, including sex, age, stroke onset time, stroke onset season (Spring for March to May, Summer for June to August, Autumn for September to November, and Winter for December to February), and medical history (hypertension, diabetes mellitus, smoke, drink, atrial fibrillation, previous history of stroke and coronary heart disease). Laboratory data included four items of blood coagulation (prothrombin time, fibrinogen, activated partial thromboplastin time, and thrombin time) and D-Dimer. Imaging data included the Alberta Stroke Program Early CT Score (ASPECTS) or posterior circulation Alberta Stroke Program Early CT Score (pc-ASPECTS) assessed on initial CT and malignant brain edema and hemorrhagic transformation (HT) assessed on review CT. Clinical features included admission and discharge National Institutes of Health Stroke Scale (NIHSS) scores, change in NIHSS score (ΔNIHSS: discharge NIHSS score minus admission NIHSS score), stroke subtypes, time from onset to EVT, and early vascular recanalization. Stroke subtypes were estimated by an experienced neurologist and are classified into three categories (cardioembolic stroke, large-artery atherosclerosis stroke, and other types of stroke) according to the Trial of Org 10172 in Acute Stroke Treatment criteria (14).

### 2.3 Outcome assessments

We explored the following primary outcomes based on NIHSS score: (1) Discharge severity of stroke, as measured by discharge NIHSS score (continuous variable); (2) Poor discharge prognosis, defined as discharge NIHSS ≥ 16 points (3). (3) Neurological function change, as measured by ΔNIHSS (continuous variable). (4) Neurological function deterioration, defined as ΔNIHSS ≥ 4 (15). In addition, we explored the following three secondary outcomes: (1) Malignant brain edema, defined as the presence of large hypodensity lesions at hemisphere causing midline shift >5 mm on CT review or need for decompressive hemicraniectomy or death (16); (2) HT evaluated on the 24-h review CT scan image; (3) Early vascular recanalization, described as modified Thrombolysis in Cerebral Infarction Score (mTICI) ≥ 2b.

### 2.4 Statistical analysis

Continuous variables were expressed as mean ± SD or median (IQR) based on their distribution and compared using Student *T*-test or Mann–Whitney U-test. Categorical variables were summarized as count (percentage) and compared using the χ^2^ test or Fisher's exact test. To assess the association of stroke onset time [day-onset (6:00 h−18:00 h) vs. night-onset (18:00 h−6:00 h) groups], multivariate linear regression was used for continuous outcome variables (discharge NIHSS score and ΔNIHSS score), and multivariate logistic regression was used for binary outcome variables (discharge NIHSS ≥16, ΔNIHSS ≥4, malignant brain edema, hemorrhagic transformation, and early vascular recanalization). We conducted not only extensive assessments in the overall cohort, but also subgroup analyses in different age groups (< 55 y, 55–64 y, 65–74 y, and 75+ y). Age, sex, hypertension, diabetes mellitus, smoking, alcohol consumption, atrial fibrillation, previous history of stroke, coronary heart disease, onset to surgery time, and stroke onset season were added into the models as covariates.

Point-Biserial Correlation analysis was performed to investigate the correlation between discharge NIHSS score and malignant brain edema, HT, and early vascular recanalization. In addition, mediation models were developed to explore the role of malignant brain edema, HT, and early vascular recanalization on the association between onset time and discharge NIHSS score. Indirect effects were estimated with bias-corrected (BC) bootstrapping using 1,000 iterations. All analyses were performed using SPSS (version 25.0; IBM, Armonk, NY, USA) and Amos (version 26.0.0; IBM, Armonk, NY, USA) software. P < 0.05 was considered statistically significant. The significance of the BC bootstrap estimate was indicated by confidence intervals (CI) that did not contain 0 (17).

## 3 Results

### 3.1 Baseline characteristics of the study population

Table 1 summarizes the demographic and clinical characteristics of the overall cohort divided into day and night groups. The mean (±SD) age of stroke patients was 68.05 (±12.31) years, and 331 cases were men (70.4%). Patients who occurred stroke at night (18:00 h−6:00 h) were younger (*P* = 0.037), had a higher proportion of a history of atrial fibrillation (*P* = 0.041), and had longer onset to EVT time (*P* < 0.001). Other baseline variables were not significantly associated with stroke onset time. Here, we observed a circadian dependence in the stroke onset age. Although the age difference between the day-onset and night-onset groups was statistically significant, the absolute difference of 2.4 years is still of uncertain clinical relevance in the context of stroke outcomes.

**Table 1:** Demographic and clinical characteristics of total patients.

**Characteristics**	**All patients (*n =* 470)**	**6:00–18:00 (*n =* 275)**	**18:00–6:00 (*n =* 195)**	***P*-value**
**Demographic characteristics**				
Gender, *n* (%)				0.056
Male	331 (70.4%)	203 (73.8%)	128 (65.6%)	
Female	139 (29.6%)	72 (26.2%)	67 (34.4%)	
Age, mean ± SD	68.05 ± 12.31	69.04 ± 12.16	66.64 ± 12.42	0.037^*^
**Stroke onset season**, ***n*** **(%)**				0.992
Spring	119 (25.3%)	70 (25.5%)	49 (25.1%)	
Summer	127 (27.0%)	75 (27.3%)	52 (26.6%)	
Autumn	107 (22.8%)	63 (22.9%)	44 (22.6%)	
Winter	117 (24.9%)	67 (24.4%)	50 (25.6%)	
**Stroke subtype**, ***n*** **(%)**				0.749
Cardioembolism	134 (28.5%)	82 (29.8%)	52 (26.6%)	
Large-artery atherosclerosis	116 (24.7%)	66 (24.0%)	50 (25.6%)	
Other subtypes	220 (46.8%)	127 (46.2%)	93 (47.7%)	
**Medical history**, ***n*** **(%)**				
Hypertension	299 (63.6%)	177 (64.4%)	122 (62.6%)	0.689
Diabetes mellitus	101 (21.5%)	57 (20.7%)	44 (22.6%)	0.633
Smoke	178 (37.9%)	110 (40.0%)	68 (34.9%)	0.259
Drink	156 (33.2%)	91 (33.1%)	65 (33.3%)	0.956
Atrial fibrillation	108 (23.0%)	54 (19.6%)	54 (27.7%)	0.041^*^
Previous History of Stroke	46 (9.8%)	27 (9.8%)	19 (9.7%)	0.979
Coronary heart disease	46 (9.8%)	30 (10.9%)	16 (8.2%)	0.331
**Radiological investigations**				
ASPECT/pc-ASPECTS, median (IQR)	8.0 (3.0)	8.0 (3.0)	8.0 (3.0)	0.957
Malignant brain edema, *n* (%)	89 (19.0%)	51 (18.5%)	38 (19.5%)	0.827
Hemorrhagic transformation, *n* (%)	181 (38.5%)	111 (40.4%)	70 (35.9%)	0.327
**Clinical characteristics**				
NIHSS on admission as continuous, mean ± SD	15.84 ± 7.73	16.09 ± 7.98	15.50 ± 3.78	0.420
NIHSS on admission ≥ 16, *n* (%)	223 (47.4%)	136 (49.5%)	87 (44.6%)	0.283
Onset to surgery time(min), median (IQR)	408.5 (202.5)	396.0 (160.0)	432.0 (358.0)	< 0.001^*^
Early vascular recanalization(mTICI≥2b), *n* (%)	391 (83.2%)	228 (82.9%)	163 (83.6%)	0.884
NIHSS on discharge as continuous, median (IQR)	10.0 (18.0)	10.0 (21.0)	10.0 (15.0)	0.669
NIHSS on discharge ≥ 16, *n* (%)	152 (32.3%)	91 (33.1%)	61 (31.3%)	0.680
ΔNIHSS as continuous, mean ± SD	1.19 ± 13.06	0.96 ± 13.46	1.50 ± 12.50	0.663
ΔNIHSS ≥ 4, *n* (%)	114 (24.3%)	71 (25.8%)	43 (22.1%)	0.337
**Laboratory data**				
Prothrombin time(s), mean ± SD	14.45 ± 2.02	14.41 ± 1.85	14.51 ± 2.25	0.636
Fibrinogen(g/L), mean ± SD	2.84 ± 0.92	2.78 ± 0.90	2.93 ± 0.94	0.085
Activated partial thromboplastin time(s), mean ± SD	45.33 ± 21.26	34.30 ± 6.02	34.58 ± 5.58	0.747
Thrombin time(s), median (IQR)	18.3 (6.6)	18.6 (6.6)	17.8 (6.5)	0.349
D-Dimer(mg/L), median (IQR)	1.4 (2.3)	1.5 (3.0)	1.2 (1.8)	0.083

NIHSS, National Institutes of Health Stroke Scale; ASPECTS, Alberta Stroke Program Early CT score; pc-ASPECTS, posterior circulation Alberta Stroke Program Early CT Score; ΔNIHSS, discharge minus admission NIHSS score; mTICI, modified thrombolysis in cerebral infarction.

^*^Significant P-value.

Thereupon, we performed a subgroup analysis dividing patients into four groups based on stroke onset age: < 55 y (*n* = 68), 55-64 y (*n* = 90), 65-74 y (*n* = 156), and 75+ y (*n* = 156). For each age group, we further divided day and night stroke onset time groups and conducted univariate analysis. Except for AIS patients younger than 55 years of age, the age of patients presenting with nighttime strokes is significantly different than those presenting with daytime strokes across the other three groups (Supplementary Tables 1–3). In patients aged 55–64, 65–74, and 75+ y, there were more males than females, while in the group aged < 55 y, there were relatively equal numbers of males and females (Supplementary Figure 2). There were 68 patients younger than 55 years old, of which 37 (54.41%) were day-onset cases and 31 (45.59%) were night-onset. Patients who develop stroke at night were more likely to have a history of atrial fibrillation (*P* = 0.016). Among the day-onset patients, 12 cases (32.4%) had malignant brain edema (*P* = 0.024), 18 cases (48.6%) had HT (*P* = 0.026), 14 cases (37.8%) had discharge NIHSS score ≥16 (*P* = 0.020), and 13 cases (35.1%) had ΔNIHSS ≥ 4 (*P* = 0.014) (Table 2), suggesting that day-onset stroke patients younger than 55 years of age showed notable circadian rhythms in discharge outcomes, neurological deterioration, malignant brain edema, and HT. Whereas in the other three age groups, there was no significant difference (Supplementary Tables 1–3).

**Table 2:** Demographic and clinical characteristics of patients with age < 55 years.

**Characteristics**	** < 55 years patients (*n =* 68)**	**6:00–18:00 (*n =* 37)**	**18:00–6:00 (*n =* 31)**	***P*-value**
**Demographic characteristics**				
Gender, *n* (%)				0.992
Male	37 (54.4%)	31 (83.8%)	6 (19.4%)	
Female	31 (45.6%)	26 (70.3%)	5 (16.1%)	
Age, mean ± SD	46.12 ± 6.41	47.38 ± 5.72	44.61 ± 6.95	0.076
**Stroke onset season**, ***n*** **(%)**				0.090
Spring	17 (25.0%)	6 (16.2%)	11 (35.5%)	
Summer	19 (27.9%)	10 (27.0%)	9 (29.0%)	
Autumn	15 (22.1%)	12 (32.4%)	3 (9.7%)	
Winter	17 (25.0%)	9 (24.3%)	8 (25.8%)	
**Stroke subtype**, ***n*** **(%)**				0.581
Cardioembolism	13 (19.1%)	8 (21.6%)	5 (16.1%)	
Large-artery atherosclerosis	18 (26.5%)	11 (29.7%)	7 (22.6%)	
Other subtypes	37 (54.4%)	18 (48.6%)	19 (61.3%)	
**Medical history**, ***n*** **(%)**				
Hypertension	32 (47.1%)	16 (43.2%)	16 (51.6%)	0.491
Diabetes mellitus	7 (10.3%)	4 (10.8%)	3 (9.7%)	0.600
Smoke	36 (52.9%)	20 (54.1%)	16 (51.6%)	0.841
Drink	29 (42.6%)	13 (35.1%)	16 (51.6%)	0.171
Atrial fibrillation	5 (7.4%)	0 (0.0%)	5 (16.1%)	0.016^*^
Previous History of Stroke	2 (2.9%)	2 (5.4%)	0 (0.0%)	0.496
Coronary heart disease	1 (1.5%)	1 (2.7%)	0 (0.0%)	1.000
**Radiological investigations**				
ASPECT/pc-ASPECTS, median (IQR)	7.0 (3.0)	7.0 (2.5)	8.0 (2.0)	0.249
Malignant brain edema, *n* (%)	15 (22.1%)	12 (32.4%)	3 (9.7%)	0.024^*^
Hemorrhagic transformation, *n* (%)	25 (36.8%)	18 (48.6%)	7 (22.6%)	0.026^*^
**Clinical characteristics**				
NIHSS on admission as continuous, mean ± SD	12.88 ± 6.10	13.14 ± 6.66	12.58 ± 5.45	0.712
NIHSS on admission ≥ 16, *n* (%)	21 (30.9%)	15 (40.5%)	6 (19.4%)	0.060
Onset to surgery time(min), median (IQR)	400.5 (220.3)	414.0 (203.5)	392.0 (261.0)	0.868
Early vascular recanalization(mTICI≥2b), *n* (%)	55 (80.9%)	27 (73.0%)	28 (90.3%)	0.070
NIHSS on discharge as continuous, median (IQR)	8.0 (12.8)	11.0 (27.5)	7.0 (8.0)	0.053
NIHSS on discharge ≥ 16, *n* (%)	18 (26.5%)	14 (37.8%)	4 (12.9%)	0.020^*^
ΔNIHSS as continuous, median (IQR)	3.0 (8.8)	2.0 (22.0)	5.0 (8.0)	0.063
ΔNIHSS ≥ 4, *n* (%)	16 (23.5%)	13 (35.1%)	3 (9.7%)	0.014^*^
**Laboratory data**				
Prothrombin time(s), mean ± SD	14.22 ± 1.88	14.04 ± 1.27	14.41 ± 2.41	0.427
Fibrinogen(g/L), median (IQR)	2.6 (0.8)	2.5 (0.6)	2.6 (1.5)	0.295
Activated partial thromboplastin time(s), mean ± SD	42.62 ± 23.13	19.93 ± 7.96	25.12 ± 23.88	0.699
Thrombin time(s), median (IQR)	17.5 (4.53)	17.4 (4.8)	18.2 (3.9)	0.717
D-Dimer(mg/L), median (IQR)	1.80 ± 2.97	1.77 ± 2.42	1.84 ± 3.49	0.926

NIHSS, National Institutes of Health Stroke Scale; ASPECTS, Alberta Stroke Program Early CT score; pc-ASPECTS, posterior circulation Alberta Stroke Program Early CT Score; ΔNIHSS, discharge minus admission NIHSS score; mTICI, modified thrombolysis in cerebral infarction.

^*^Significant P-value.

### 3.2 Associations of stroke onset time with discharge outcomes and neurological deterioration in patients younger than 55 years of age

We performed multivariable linear and logistic regression analysis for patients in different age groups. After adjusting for age, sex, hypertension, diabetes mellitus, smoking, alcohol consumption, atrial fibrillation, previous history of stroke, coronary heart disease, onset to EVT time, and stroke onset season, stroke onset time was independently associated with discharge NIHSS ≥ 16 (adjusted OR = 0.136, 95%CI = 0.027–0.678, *P* = 0.015), ΔNIHSS ≥ 4 (adjusted OR = 0.081, 95%CI = 0.012–0.544, *P* = 0.010), malignant brain edema (adjusted OR = 0.145, 95%CI = 0.027–0.798, *P* = 0.026), HT (adjusted OR = 0.231, 95%CI = 0.057–0.946, *P* = 0.042), early vascular recanalization (adjusted OR = 0.118, 95%CI = 0.017–0.813, *P* = 0.007) (Table 3) and discharge NIHSS score (standard β = −0.334, 95%CI: −0.021, −0.005, *P* = 0.003), ΔNIHSS score (standard β = 0.343, 95%CI = 0.005–0.022, *P* = 0.002) (Table 4). These results suggested that night-onset stroke had a better discharge prognosis and was less likely to suffer from neurological deterioration, malignant brain edema, and HT, and more likely to occur with early vascular recanalization.

**Table 3:** Multivariable logistic regression for associations between outcomes and stroke onset time of patients with age < 55 years.

**Characteristics**	**Adjusted OR (95% CI)**	**Adjusted P-value**
NIHSS on discharge≥16	0.136 (0.027–0.678)	0.015^*^
ΔNIHSS≥4	0.081 (0.012–0.544)	0.010^*^
Malignant brain edema	0.145 (0.027–0.798)	0.026^*^
Hemorrhagic transformation	0.231 (0.057–0.946)	0.042^*^
Early vascular recanalization(mTICI≥2b)	0.118 (0.017–0.813)	0.007^*^

Adjusted for: Age, Gender, Hypertension, Diabetes mellitus, Smoking, Alcohol consumption, Atrial fibrillation, Previous history of stroke, Coronary heart disease, Onset to surgery time, Stroke onset season.

NIHSS, National Institutes of Health Stroke Scale; ΔNIHSS, discharge minus admission NIHSS score; mTICI, modified thrombolysis in cerebral infarction.

^*^Significant P-value.

**Table 4:** Multivariable linear regression for associations between continuous outcomes and stroke onset time of patients with age < 55 years.

**Characteristics**	**Standard β (95% CI)**	**Adjusted *P*-value**
NIHSS on discharge as continuous	−0.334 (−0.021 to −0.005)	0.003^*^
ΔNIHSS as continuous	0.343 (0.005–0.022)	0.002^*^

Adjusted for: Age, Gender, Hypertension, Diabetes mellitus, Smoking, Alcohol consumption, Atrial fibrillation, Previous history of stroke, Coronary heart disease, Onset to surgery time, Stroke onset season.

NIHSS, National Institutes of Health Stroke Scale; ΔNIHSS, discharge minus admission NIHSS score.

^*^Significant P-value.

**Figure 1:**
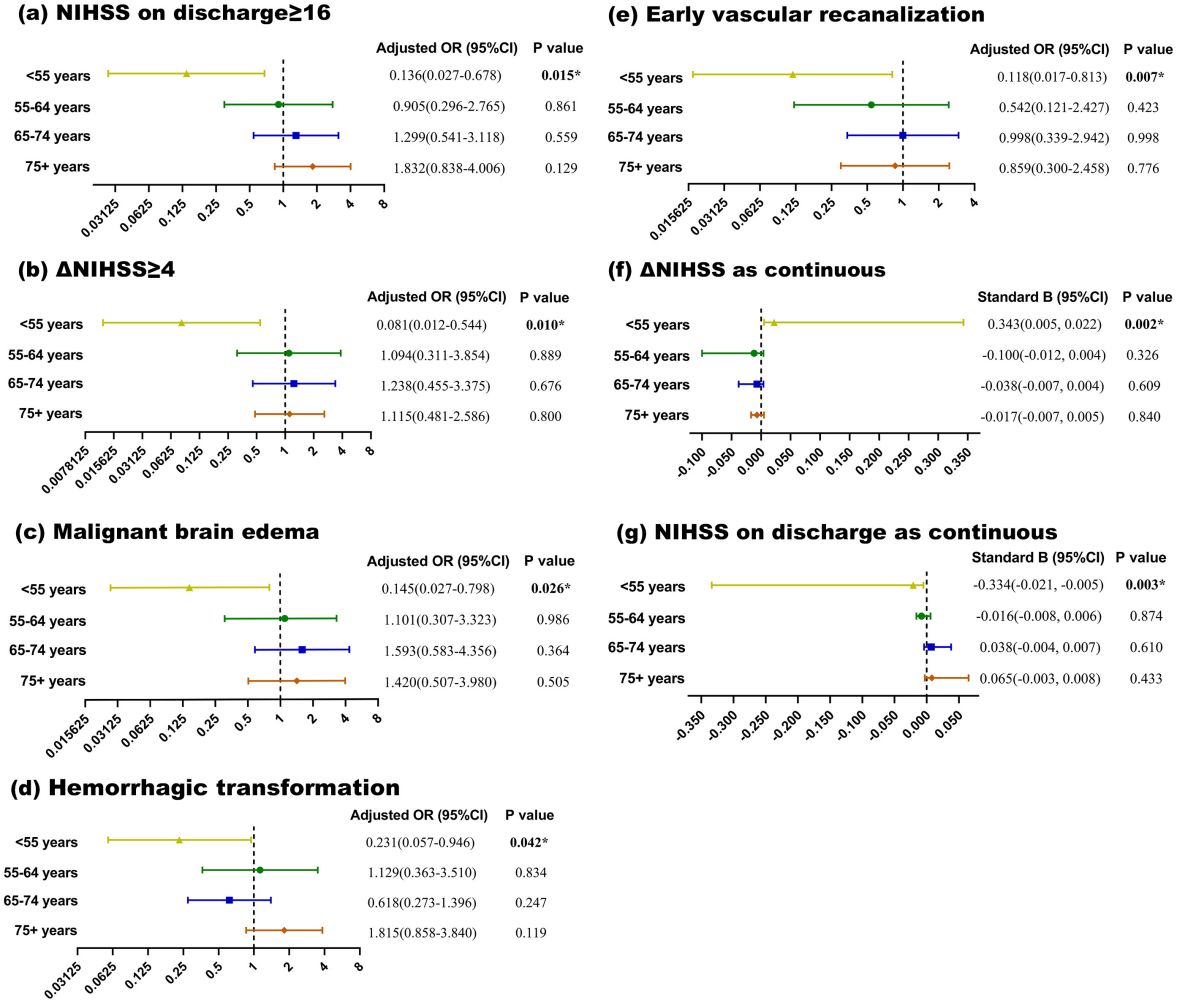
Multivariable and linear logistic regression results of outcomes in different age groups. **(a–e)** respectively represent multivariable logistic regression results of NIHSS on discharge≥16, ΔNIHSS≥4, malignant brain edema, hemorrhagic transformation and early vascular recanalization in different age groups. **(f, g)** Respectively represent multivariable linear regression results of NIHSS on discharge as continuous and ΔNIHSS as continuous. Adjusted for: Age, Sex, Hypertension, Diabetes mellitus, Smoking, Alcohol consumption, Atrial fibrillation, Previous history of stroke, Coronary heart disease, Onset to EVT time, Stroke onset season. NIHSS, National Institutes of Health Stroke Scale; ΔNIHSS, discharge minus admission NIHSS score. *Significant *P*-value.

Figure 1 shows the comparison of multivariate linear and logistic regression results of different outcome variables between different age groups. As illustrated, only patients with age < 55 y showed circadian difference in functional outcomes, while in the other three age groups there was no independent correlation between functional outcomes and stroke onset time.

### 3.3 Point-Biserial Correlation analysis and mediation analysis in patients younger than 55 years of age

The results of Point-Biserial Correlation analysis between discharge NIHSS score and malignant brain edema, HT, and early vascular recanalization are shown in Table 5. Discharge NIHSS score was positively associated with malignant brain edema (*r* = 0.358, *P* = 0.003) and HT (*r* = 0.336, *P* = 0.005), while there was no significant correlation between discharge NIHSS score and early vascular recanalization.

**Table 5:** Point-Biserial Correlation analysis of NIHSS on discharge as continuous with Malignant brain edema, Hemorrhagic transformation and Early vascular recanalization (mTICI ≥ 2b) of patients with age < 55 years.

**Characteristics**	**NIHSS on discharge as continuous**
Malignant brain edema	*r =* 0.345, *P =* 0.004^*^
Hemorrhagic transformation	*r =* 0.302, *P =* 0.012^*^
Early vascular recanalization (mTICI ≥ 2b)	*r =* −0.228, *P =* 0.062

NIHSS, National Institutes of Health Stroke Scale; mTICI, modified thrombolysis in cerebral infarction.

^*^Significant P-value.

**Figure 2:**
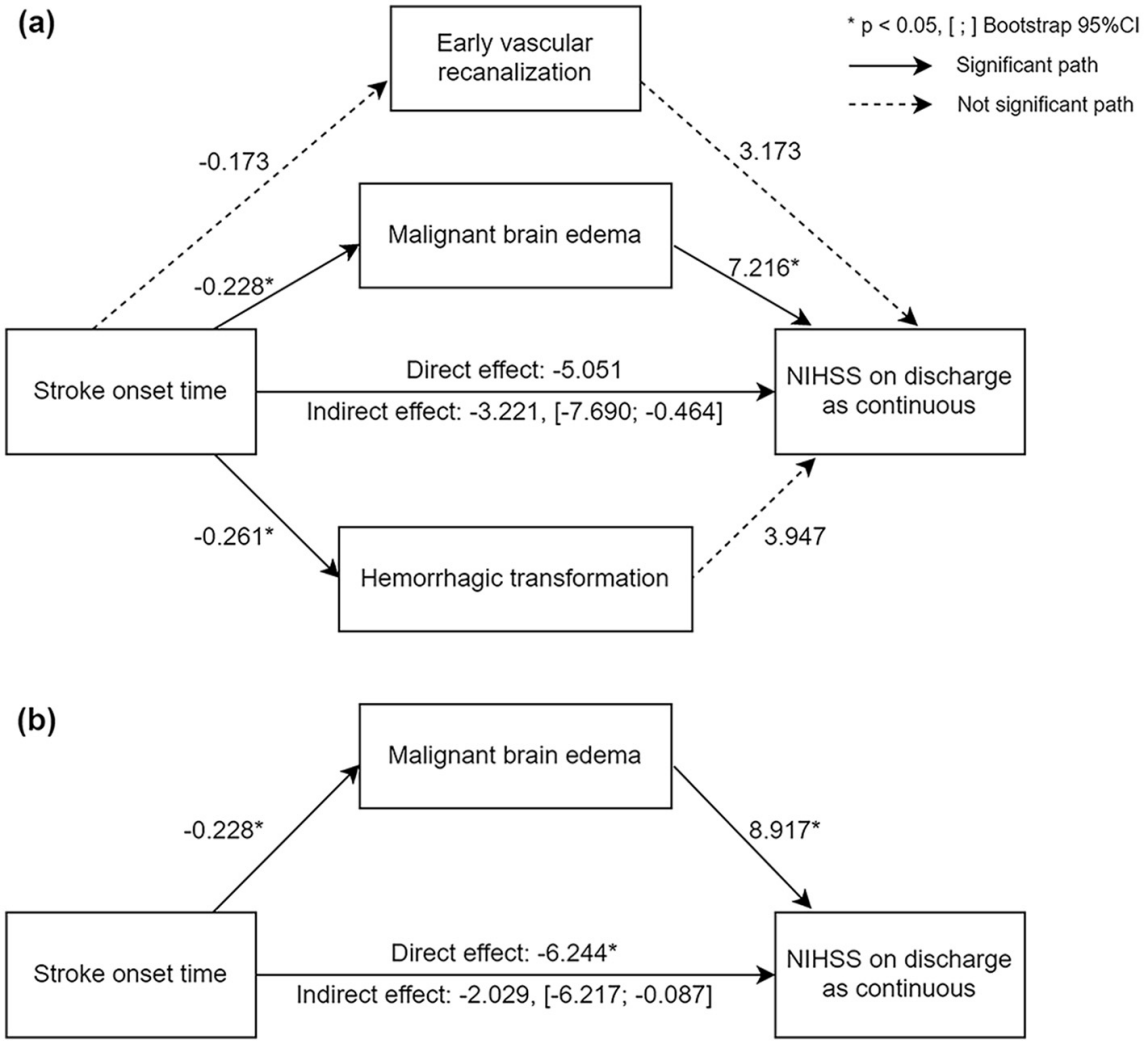
Diagram of mediation analyses with pathways from stroke onset time to discharge NIHSS score. **(a)** Parallel mediation model; **(b)** simple mediation model. The number represents the regression coefficient for each path. Solid lines represent significant pathway and dotted lines represent not significant pathways. *Significant *P*-value. [;], The 95%CI of indirect effect validated by bootstrap test.

Mediation model was established to investigate whether the association between stroke onset time and discharge NIHSS score was mediated by malignant brain edema, HT, or early vascular recanalization. With stroke onset time as the independent variable, discharge NIHSS score as the dependent variable, and malignant brain edema, HT, and early vascular recanalization as mediators, we found that the mediation effect was significant (*c*' = −3.221, BC 95%CI = −7.690; −0.464), in which only the pathway stroke onset time → malignant brain edema → discharge NIHSS score showed significant result (Figure 2). Therefore, HT and early vascular recanalization were removed from the model, and we established a simple mediation model of stroke onset time → malignant brain edema → discharge NIHSS score. The result showed that the indirect effect of malignant brain edema was significant (*c*' = −2.029, BC 95%CI = −6.217; −0.087) (Figure 2), implying that the correlation of stroke onset time and stroke prognosis was mediated by malignant brain edema.

## 4 Discussion

In this study, we observed that: (1) Younger patients showed more significant diurnal-night differences in the outcomes than the elderly; (2) There were diurnal-night differences in discharge prognosis in younger (< 55 y) patients, the prognosis of night-onset was better than that of day-onset; (3) The circadian difference of stroke prognosis may be related to the diurnal difference of malignant brain edema. This is basically consistent with our original assumption.

In a recent study, Benali et al. (18) revealed this phenomenon: based on the findings that AIS patients treated with EVT at night were more likely to have a clinically favorable 3-month outcome, the author further compared the characteristics of patients treated at night with favorable vs. unfavorable outcomes and found no significant differences in other medical history characteristics other than age. Age is the most considerable risk factor for AIS, as it influence the outcome among AIS patients and can't be modified (19). Jayaraman et al. (20) quantified the relationship between age and prognosis after EVT and found that among recanalized patients, modified Rankin Scale (mRS) worsened with the increase of age. In our study, no circadian rhythm difference was found for AIS outcomes across the whole cohort. However, we also detected significant age differences between day-onset and night-onset patients. Similar to the previous study (18), we found that night-onset stroke patients were slightly younger than those with day-onset stroke. Therefore, we further performed a subgroup analysis based on stroke onset age. In addition to AIS patients younger than 55 y, there also existed a significant difference in the stroke onset age in the other three groups. We found that there were diurnal-night differences in discharge prognosis in younger (< 55 y) patients, the prognosis of night-onset was better than that of day-onset, while no circadian rhythm difference was found in the elderly. This suggested that age of onset may influence circadian differences in the outcomes of AIS patients treated with EVT.

The possible reason was that circadian rhythm is dynamic across lifetimes, with younger people having stronger circadian rhythms than older people. Recent animal studies have shown that glial expression of the clock protein PERIOD decreases with age in fly models (21) and drpr mRNA showed a rhythmic profile only in young flies compared with the older (22). Similarly, a recent human study showed that rhythmic programs of mRNA generally dampened with age in most tissues, which means that younger people have stronger rhythmic programs than older people (23). In addition, age-related changes in circadian rhythm may cause pathological changes, such as chronic inflammation and metabolic disorders (24). As a result, no diurnal difference in stroke prognosis was observed in older patients in our study. More attention should be paid to the effect of patient age on circadian rhythm in future stroke treatment and management. Moreover, the majority of animal studies on stroke used young-adult rodents, though stroke occurs mainly in the aged human population (25). The findings of this study provided a certain reference for the selection of animals for research purposes. Age may have been neglected in rodent models of stroke, which might be the reason why the translation of rodent models to human stroke was found difficult.

Moreover, we found that the circadian difference in stroke outcomes in young stroke patients may be related to the diurnal difference in malignant brain edema, HT, and early vascular recanalization. Among them, malignant brain edema mediated the association of circadian rhythm and the outcome of young stroke patients. Brain edema is one of the severe complications closely related to poor outcomes of stroke (26). Despite no circadian rhythm differences have been found in previously published studies (3, 6, 27), in this study, we observed circadian rhythm differences in malignant brain edema in young patients, which was more likely to occur during the day. We speculated that the underlying reasons might be related to the rhythmic changes in the blood-brain barrier (BBB) permeability and the glymphatic function. A recent animal study showed increasing BBB permeability during the active phase and decreasing BBB permeability during the resting phase of mice (28). Hence, we infer that the BBB permeability increased during diurnal active period, which ultimately increased the incidence of malignant brain edema after stroke. Whereas, the BBB permeability decreased during the night when the BBB rhythm is more stable, and therefore the probability of malignant brain edema during the night was lower. The glymphatic system also plays a vital role during the formation of brain edema, promoting the clearance of vascular edema in the late stage (29). A previous study showed that glymphatic clearance exhibit endogenous circadian rhythms, which was enhanced during sleep and inhibited during wakefulness period (30). This was consistent with our result that malignant brain edema was more likely to occur during the day. In contrast, in older adults, the nocturnal circadian rhythms were more erratic, hence no difference was observed. This speculation needs to be verified by further clinical and experimental studies in the future.

We acknowledged some limitations of our study. First, the proportion of patients in the younger age group (< 55 y) was relatively small, which may cause experimental occasion. Second, this is a retrospective study based on EVT, which may lead to possible selection bias. Third, due to the lack of data, we used the NIHSS score as the outcome measure instead of the mRS Score, which may be more representative. Last but not least, a key finding of our univariate analysis was that patients with night-onset stroke were significantly younger than those with day-onset stroke. However, this difference did not survive correction for multiple testing (Bonferroni correction). This highlights an important limitation of our study. The observed differences in Table 1, while intriguing and potentially clinically relevant (e.g., the 2.4-year age difference), must be interpreted with extreme caution as they may represent false positive findings. They should be considered as generating hypotheses rather than confirming associations. To address these limitations, future studies with larger sample sizes and *a priori* hypotheses are needed to validate whether the conclusions.

In conclusion, the onset age could influence the circadian difference in the outcome of AIS patients treated with EVT. The prognosis of young stroke patients (< 55 y) treated with EVT showed diurnal difference, night-onset patients showed better outcomes compared with day-onset patients, which may be related to the diurnal difference in malignant brain edema. However, these results were related to a small subgroup of patients and given the methodological statistical limitations (multiple testing correction), these results are only driving hypothesis. Further research with larger patient sizes are required to validate these results and explore the underlying mechanisms. More attention should be paid to the patient's age on the effect of circadian rhythm in future stroke treatment and management.

## Data Availability

The datasets presented in this article are not readily available because they are private data. Requests to access the datasets should be directed to wyyyfskxnz@163.com.

## References

[1] Lo EH Albers GW Dichgans M Donnan G Esposito E Foster R. Circadian biology and stroke. Stroke. (2021) 52:2180–90. 10.1161/STROKEAHA.120.031742 33940951

[2] Esposito E Li W Mandeville TE Park J-H Sencan I Guo S. Potential circadian effects on translational failure for neuroprotection. Nature. (2020) 582:395–8. 10.1038/s41586-020-2348-z 32494010 PMC9466001

[3] Wang X Wang XY Ma J Jia ML Wu LF Li WL. Association between the time of day at stroke onset and functional outcome of acute ischemic stroke patients treated with endovascular therapy. J Cerebral Blood Flow Metab. (2022) 42:2191–200. 10.1177/0271678X221111852 35791272 PMC9670006

[4] Ryu WS Hong KS Jeong SW Park JE Kim BJ Kim JT. Association of ischemic stroke onset time with presenting severity, acute progression, and long-term outcome: a cohort study. PLoS Med. (2022) 19:e1003910. 10.1371/journal.pmed.1003910 35120123 PMC8815976

[5] Yu S-Y Sun Q Chen S-N Wang F Chen R Chen J. Circadian rhythm disturbance in acute ischemic stroke patients and its effect on prognosis. Cerebrovasc. Dis. (2024) 53, 14–27. 10.1159/000528724 37423205

[6] Ding J Bai Z Zhou D Li X Rajah GB Ding Y. Circadian rhythms may not influence the outcomes of thrombolysis in patients with ischemic stroke: A study from China. Chronobiol Int. (2018) 35:1533–42. 10.1080/07420528.2018.1494602 29993298

[7] Collaborators GBDS. Global, regional, and national burden of stroke and its risk factors, 1990-2019: a systematic analysis for the Global Burden of Disease Study 2019. Lancet Neurol. (2021) 20:795–820. 10.1016/S1474-4422(21)00252-0 34487721 PMC8443449

[8] Smajlovic D. Strokes in young adults: epidemiology and prevention. Vasc Health Risk Manag. (2015) 11:157–64. 10.2147/VHRM.S53203 25750539 PMC4348138

[9] Bejot Y Daubail B Jacquin A Durier J Osseby GV Rouaud O. Trends in the incidence of ischaemic stroke in young adults between 1985 and 2011: the Dijon Stroke Registry. J Neurol Neurosurg Psychiatry. (2014) 85:509–13. 10.1136/jnnp-2013-306203 24249786

[10] Li L Scott CA Rothwell PM. Association of younger vs older ages with changes in incidence of stroke and other vascular events, 2002–2018. JAMA. (2022) 328:563–74. 10.1001/jama.2022.12759 35943470 PMC9364129

[11] Ohya Y Matsuo R Sato N Irie F Nakamura K Wakisaka Y. Causes of ischemic stroke in young adults vs. non-young adults: A multicenter hospital-based observational study. PLoS ONE. (2022) 17:e0268481. 10.1371/journal.pone.0268481 35830430 PMC9278748

[12] Hood S Amir S. The aging clock: circadian rhythms and later life. J Clin Invest. (2017) 127:437–46. 10.1172/JCI90328 28145903 PMC5272178

[13] Talamanca L Gobet C Naef F. Sex-dimorphic and age-dependent organization of 24-hour gene expression rhythms in humans. Science. (2023) 379:478–83. 10.1126/science.add0846 36730411

[14] The Publications Committee for the Trial of ORG 10172 in Acute Stroke Treatment (TOAST) Investigators. Low molecular weight heparinoid, ORG 10172 (danaparoid), and outcome after acute ischemic stroke. JAMA. (1998) 279, 1265–1272. 10.1001/jama.279.16.1265 9565006

[15] Saleem Y Nogueira RG Rodrigues GM Kim S Sharashidze V Frankel M. Acute neurological deterioration in large vessel occlusions and mild symptoms managed medically. Stroke. (2020) 51:1428–34. 10.1161/STROKEAHA.119.027011 32295503

[16] Ong CJ Gluckstein J Laurido-Soto O Yan Y Dhar R Lee J-M. Enhanced detection of Edema in Malignant Anterior Circulation Stroke (EDEMA) score. Stroke. (2017) 48, 1969–72. 10.1161/STROKEAHA.117.016733 28487333 PMC5487281

[17] Shrout PE Bolger N. Mediation in experimental and nonexperimental studies: New procedures and recommendations. Psychol Methods. (2002) 7:422–45. 10.1037/1082-989X.7.4.422 12530702

[18] Benali A Moynier M Dargazanli C Deverdun J Cagnazzo F Mourand I. Mechanical thrombectomy in nighttime hours: is there a difference in 90-day clinical outcome for patients with ischemic stroke? AJNR Am J Neuroradiol. (2021) 42:530–7. 10.3174/ajnr.A6997 33478943 PMC7959420

[19] Roy-O'Reilly M McCullough LD. Age and sex are critical factors in ischemic stroke pathology. Endocrinology. (2018) 159:3120–31. 10.1210/en.2018-00465 30010821 PMC6963709

[20] Jayaraman MV Kishkovich T Baird GL Hemendinger ML Tung EL Yaghi S. Association between age and outcomes following thrombectomy for anterior circulation emergent large vessel occlusion is determined by degree of recanalisation. J Neurointerv Surg. (2019) 11:114–8. 10.1136/neurintsurg-2018-013964 29858396

[21] Long DM Giebultowicz JM. Age-related changes in the expression of the circadian clock protein PERIOD in drosophila glial cells. Front Physiol. (2017) 8:1131. 10.3389/fphys.2017.01131 29375400 PMC5767304

[22] Kuintzle RC Chow ES Westby TN Gvakharia BO Giebultowicz JM Hendrix DA. Circadian deep sequencing reveals stress-response genes that adopt robust rhythmic expression during aging. Nat Commun. (2017) 8:14529. 10.1038/ncomms14529 28221375 PMC5321795

[23] Barth E Srivastava A Wengerodt D Stojiljkovic M Axer H Witte OW. Age-dependent expression changes of circadian system-related genes reveal a potentially conserved link to aging. Aging. (2021) 13:25694–716. 10.18632/aging.203788 34923482 PMC8751596

[24] Khan S Siddique R Liu Y Yong VW Xue M. Towards improving the prognosis of stroke through targeting the circadian clock system. Int J Biol Sci. (2024) 20:403–13. 10.7150/ijbs.88370 38169640 PMC10758097

[25] Zhang H Lin S Chen X Gu L Zhu X Zhang Y. The effect of age, sex and strains on the performance and outcome in animal models of stroke. Neurochem Int. (2019) 127:2–11. 10.1016/j.neuint.2018.10.005 30291954

[26] Chen S Shao L Ma L. Cerebral edema formation after stroke: emphasis on blood–brain barrier and the lymphatic drainage system of the brain. Front Cell Neurosci. (2021) 15:716825. 10.3389/fncel.2021.716825 34483842 PMC8415457

[27] Lorenzano S Ahmed N Tatlisumak T Gomis M Dávalos A Mikulik R. Within-day and weekly variations of thrombolysis in acute Ischemic stroke. Stroke. (2014) 45:176–84. 10.1161/STROKEAHA.113.002133 24262329

[28] Zhang SL Lahens NF Yue Z Arnold DM Pakstis PP Schwarz JE. A circadian clock regulates efflux by the blood-brain barrier in mice and human cells. Nat Commun. (2021) 12:617. 10.1038/s41467-020-20795-9 33504784 PMC7841146

[29] Li W Chen D Liu N Luan Y Zhu S Wang H. Modulation of lymphatic transport in the central nervous system. Theranostics. (2022) 12:1117–31. 10.7150/thno.66026 35154477 PMC8771567

[30] Hablitz LM Pla V Giannetto M Vinitsky HS Staeger FF Metcalfe T. Circadian control of brain glymphatic and lymphatic fluid flow. Nat Commun. (2020) 11:4411. 10.1038/s41467-020-18115-2 32879313 PMC7468152

