# Supplementary material for: Stroke onset time affected outcomes in the young acute ischemic stroke patients treated with endovascular thrombectomy
Source: Front Neurol. 2025 Oct 23;16:1655646. doi: 10.3389/fneur.2025.1655646 (PMC12588849; doi:10.3389/fneur.2025.1655646)
Supplement: Supplementary file 1 [file Table_1.DOCX]

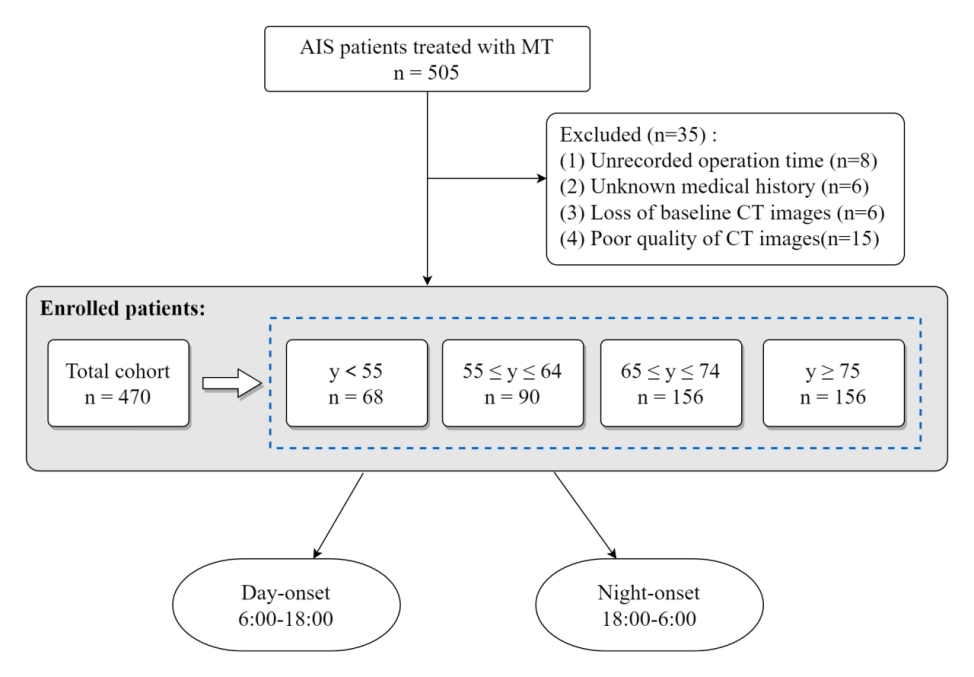


**Supplementary Figure 1. Flow chart of the study.** AIS, acute ischemic stroke; MT, mechanical thrombectomy.

|  | 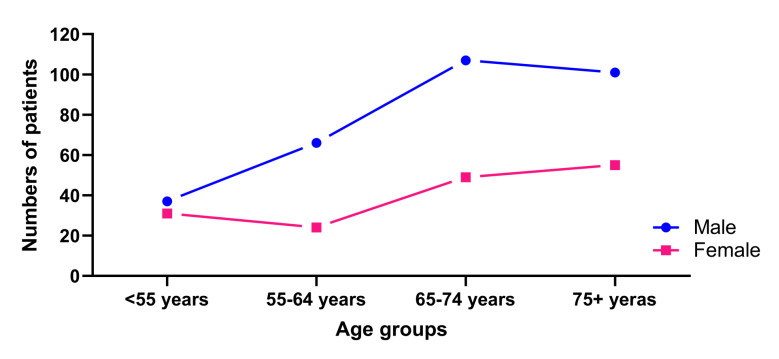 |
| --- | --- |

**Supplementary Figure 2. Gender distribution in different age groups.** Blue line for male and pink line for female. In patients aged 55-64 years, 65-74 years, and 75+ years, there were more males than females, while in the group aged < 55 years, there were relatively equal numbers of males and females.

**Supplementary Table1.** Demographic and clinical characteristics of patients with age between 55 and 64 years.

|  | 55-64 years patients (n=90) | 6:00-18:00 (n=50) | 18:00-6:00 (n=40) | P value |
| --- | --- | --- | --- | --- |
| **Demographic characteristics** |  |  |  |  |
| Gender, n (%) |  |  |  | 0.038* |
| Male | 66(73.3%) | 41(82.0%) | 25(62.5%) |  |
| Female | 24(24.7%) | 9(18.0%) | 15(37.5%) |  |
| Age, mean ± SD | 59.84±3.00 | 59.28±2.94 | 60.55±2.99 | 0.045* |
| **Stroke onset season, n (%)** |  |  |  | 0.932 |
| Spring | 20(22.2%) | 12(24.0%) | 8(20.0%) |  |
| Summer | 31(34.4%) | 16(32.0%) | 15(37.5%) |  |
| Autumn | 17(18.9%) | 10(20.0%) | 7(17.5%) |  |
| Winter | 22(24.4%) | 12(24.0%) | 10(25.0%) |  |
| **Stroke subtype, n (%)** |  |  |  | 0.710 |
| Cardioembolism | 21(23.3%) | 13(26.0%) | 8(20.0%) |  |
| Large-artery atherosclerosis | 28(31.1%) | 16(32.0%) | 12(30.0%) |  |
| Other subtypes | 41(45.6%) | 21(42.0%) | 20(50.0%) |  |
| **Medical history, n (%)** |  |  |  |  |
| Hypertension | 51(56.7%) | 29(58.0%) | 22(55.0%) | 0.775 |
| Diabetes mellitus | 22(24.4%) | 11(22.0%) | 11(27.5%) | 0.546 |
| Smoke | 36(40.0%) | 22(44.0%) | 14(35.0%) | 0.386 |
| Drink | 33(36.7%) | 17(34.0%) | 16(40.0%) | 0.557 |
| Atrial fibrillation | 21(23.3%) | 9(18.0%) | 12(30.0%) | 0.181 |
| Previous History of Stroke | 6(6.7%) | 5(10.0%) | 1(2.5%) | 0.221 |
| Coronary heart disease | 3(3.3%) | 2(4.0%) | 1(2.5%) | 1.000 |
| **Radiological investigations** |  |  |  |  |
| ASPECT/pc-ASPECTS, median (IQR) | 7.0(3.0) | 7.0(3.0) | 7.0(3.5) | 0.449 |
| Malignant brain edema, n (%) | 22(24.4%) | 11(22.0%) | 11(27.5%) | 0.546 |
| Hemorrhage transformation, n (%) | 28(31.1%) | 15(30.0%) | 13(32.5%) | 0.799 |
| **Clinical characteristics** |  |  |  |  |
| NIHSS on admission as continuous, mean ± SD | 15.10±7.80 | 16.82±8.36 | 12.95±6.51 | 0.018* |
| NIHSS on admission ≥ 16, n (%) | 42(46.7%) | 28(56.0%) | 14(35.0%) | 0.047* |
| Onset to surgery time(min), median (IQR) | 402.0(205.8) | 383.0(140.5) | 430.0(316.0) | 0.065 |
| Early recanalization(mTICI≥2b), n (%) | 75(83.3%) | 43(86.0%) | 32(80.0%) | 0.318 |
| NIHSS on discharge as continuous, mean ± SD | 13.27±14.06 | 13.90±14.88 | 12.48±13.11 | 0.635 |
| NIHSS on discharge ≥ 16, n (%) | 26(28.9%) | 16(32.0%) | 10(25.0%) | 0.467 |
| ΔNIHSS as continuous, mean ± SD | 1.83±13.10 | 2.92±13.19 | 0.48±13.00 | 0.382 |
| ΔNIHSS ≥ 4, n (%) | 19(21.1%) | 11(22.0%) | 8(20.0%) | 0.817 |
| **Laboratory data** |  |  |  |  |
| Prothrombin time(s), mean ± SD | 14.14±1.47 | 14.06±1.21 | 14.24±1.74 | 0.571 |
| Fibrinogen(g/L), mean ± SD | 2.97±1.00 | 2.89±1.13 | 3.07±0.82 | 0.408 |
| Activated partial thromboplastin time(s), mean ± SD | 46.07±22.49 | 43.57±20.42 | 49.15±24.75 | 0.258 |
| Thrombin time(s), mean ±S D | 29.56±29.26 | 29.8±28.17 | 29.25±31.13 | 0.935 |
| D-Dimer(mg/L), median (IQR) | 1.3(2.0) | 1.6(2.8) | 1.12(1.6) | 0.200 |

NIHSS: National Institutes of Health Stroke Scale; ASPECTS: Alberta Stroke Program Early CT score; pc-ASPECTS: posterior circulation Alberta Stroke Program Early CT Score; ΔNIHSS: discharge minus admission NIHSS score; mTICI: modified thrombolysis in cerebral infarction.

*Significant P value.

|  | 65-74 years patients (n=156) | | 6:00-18:00 (n=90) | 18:00-6:00 (n=66) | P value |
| --- | --- | --- | --- | --- | --- |
| **Demographic characteristics** |  | |  |  |  |
| Gender, n (%) |  | |  |  | 0.658 |
| Male | 107(68.6%) | | 63(70.0%) | 44(66.7%) |  |
| Female | 49(31.4%) | | 27(30.0%) | 22(33.3%) |  |
| Age, mean ± SD | 69.87±2.86 | | 70.29±2.77 | 69.29±2.90 | 0.030* |
| **Stroke onset season, n (%)** |  | |  |  | 0.609 |
| Spring | 40(25.6%) | | 26(28.9%) | 14(21.2%) |  |
| Summer | 44(28.2%) | | 26(28.9%) | 18(27.3%) |  |
| Autumn | 34(21.8%) | | 17(18.9%) | 17(25.8%) |  |
| Winter | 38(24.4%) | | 21(23.3%) | 17(25.8%) |  |
| **Stroke subtype, n (%)** |  | |  |  | 0.755 |
| Cardioembolism | 48(30.8%) | | 27(30.0%) | 21(31.8%) |  |
| Large-artery atherosclerosis | 39(25.0%) | | 21(23.3%) | 18(27.3%) |  |
| Other subtypes | 69(44.2%) | | 42(46.7%) | 27(40.9%) |  |
| **Medical history, n (%)** |  | |  |  |  |
| Hypertension | 102(65.4%) | | 63(70.0%) | 39(59.1%) | 0.157 |
| Diabetes mellitus | 35(22.4%) | | 22(24.4%) | 13(19.7%) | 0.483 |
| Smoke | 62(39.7%) | | 37(41.1%) | 25(37.9%) | 0.684 |
| Drink | 48(30.8%) | | 26(28.9%) | 22(33.3%) | 0.552 |
| Atrial fibrillation | 28(17.9%) | | 6(6.7%) | 22(33.3%) | <0.001* |
| Previous History of Stroke | 19(12.2%) | | 9(10.0%) | 10(15.2%) | 0.331 |
| Coronary heart disease | 28(17.9%) | | 18(20.0%) | 10(15.2%) | 0.436 |
| **Radiological investigations** |  | |  |  |  |
| ASPECT/pc-ASPECTS, median (IQR) | 8.0(3.0) | | 8.0(3.0) | 7.0(3.0) | 0.274 |
| Malignant brain edema, n (%) | 30(19.2%) | | 16(17.8%) | 14(21.2%) | 0.591 |
| Hemorrhage transformation, n (%) | 64(41.0%) | | 42(46.7%) | 22(33.3%) | 0.094 |
| **Clinical characteristics** |  | |  |  |  |
| NIHSS on admission as continuous, mean ± SD | 16.58±8.14 | | 16.73±8.73 | 16.38±7.31 | 0.791 |
| NIHSS on admission ≥ 16, n (%) | 77(49.4%) | | 41(45.6%) | 36(54.5%) | 0.297 |
| Onset to surgery time(min), median (IQR) | 405.0(208.0) | | 385.5(150.0) | 447.5(463.5) | 0.002* |
| Early recanalization(mTICI≥2b), n (%) | 128(82.1%) | | 75(83.3%) | 53(80.3%) | 0.771 |
| NIHSS on discharge as continuous, mean ± SD | 14.10±13.71 | | 14.71±14.22 | 13.27±13.04 | 0.519 |
| NIHSS on discharge ≥ 16, n (%) | 45(28.8%) | | 27(30.0%) | 18(27.3%) | 0.710 |
| ΔNIHSS as continuous, mean ± SD | 2.65±12.88 | | 2.30±12.55 | 3.11±13.41 | 0.703 |
| ΔNIHSS ≥ 4, n (%) | 31(19.9%) | | 18(20.0%) | 13(19.7%) | 0.935 |
| **Laboratory data** |  | |  |  |  |
| Prothrombin time(s), mean ± SD | 14.39±2.19 | | 14.19±1.40 | 14.76±2.98 | 0.193 |
| Fibrinogen(g/L), mean ± SD | 2.81±0.85 | | 2.78±0.84 | 2.87±0.87 | 0.508 |
| Activated partial thromboplastin time(s), mean ± SD | | 45.35±21.23 | 44.57±21.78 | 46.50±20.54 | 0.595 |
| Thrombin time(s), median (IQR) | 18.3(6.4) | | 18.5(4.4) | 18.1(15.4) | 0.719 |
| D-Dimer(mg/L), median (IQR) | 1.3(2.8) | | 1.5 (4.1) | 0.8 (1.5) | 0.059 |

**Supplementary Table2.** Demographic and clinical characteristics of patients with age between 65 and 74 years.

NIHSS: National Institutes of Health Stroke Scale; ASPECTS: Alberta Stroke Program Early CT score; pc-ASPECTS: posterior circulation Alberta Stroke Program Early CT Score; ΔNIHSS: discharge minus admission NIHSS score; mTICI: modified thrombolysis in cerebral infarction.

*Significant P value.

**Supplementary Table3.** Demographic and clinical characteristics of patients with age≥75 years.

|  | 65-74 years patients (n=156) | 6:00-18:00 (n=98) | 18:00-6:00 (n=58) | P value |
| --- | --- | --- | --- | --- |
| **Demographic characteristics** |  |  |  |  |
| Gender, n (%) |  |  |  | 0.115 |
| Male | 101(64.7%) | 68(69.4%) | 33(56.9%) |  |
| Female | 55(32.3%) | 30(30.6%) | 25(43.1%) |  |
| Age, mean ± SD | 80.52±4.17 | 81.06±4.28 | 79.6±3.84 | 0.034* |
| **Stroke onset season, n (%)** |  |  |  | 0.800 |
| Spring | 42(26.9%) | 26(26.5%) | 16(27.6%) |  |
| Summer | 33(21.2%) | 23(23.5%) | 10(17.2%) |  |
| Autumn | 41(26.3%) | 24(24.5%) | 17(29.3%) |  |
| Winter | 40(25.6%) | 25(25.5%) | 15(25.9%) |  |
| **Stroke subtype, n (%)** |  |  |  | 0.799 |
| Cardioembolism | 52(33.3%) | 34(34.7%) | 18(31.0%) |  |
| Large-artery atherosclerosis | 31(19.9%) | 18(18.4%) | 13(22.4%) |  |
| Other subtypes | 73(46.8%) | 46(46.9%) | 27(46.6%) |  |
| **Medical history, n (%)** |  |  |  |  |
| Hypertension | 114(73.1%) | 69(70.4%) | 45(77.6%) | 0.329 |
| Diabetes mellitus | 37(23.7%) | 20(20.3%) | 17(29.3%) | 0.206 |
| Smoke | 44(28.2%) | 31(31.6%) | 13(22.4%) | 0.216 |
| Drink | 46(29.5%) | 35(35.7%) | 11(19.0%) | 0.027* |
| Atrial fibrillation | 54(34.6%) | 39(39.8%) | 15(25.9%) | 0.077 |
| Previous History of Stroke | 19(12.2%) | 11(11.2%) | 8(13.8%) | 0.635 |
| Coronary heart disease | 14(9.0%) | 9(9.2%) | 5(8.6%) | 0.905 |
| **Radiological investigations** |  |  |  |  |
| ASPECT/pc-ASPECTS, median (IQR) | 8.0(2.0) | 8.0(2.0) | 8.0(3.0) | 0.969 |
| Malignant brain edema, n (%) | 22(14.1%) | 12(12.2%) | 10(17.2%) | 0.415 |
| Hemorrhage transformation, n (%) | 64(41.0%) | 36(36.7%) | 28(48.3%) | 0.157 |
| **Clinical characteristics** |  |  |  |  |
| NIHSS on admission as continuous, mean ± SD | 16.83±7.63 | 16.24±7.36 | 17.83±8.02 | 0.211 |
| NIHSS on admission ≥ 16, n (%) | 83(53.2%) | 52(53.1%) | 31(53.4%) | 0.963 |
| Onset to surgery time(min), median (IQR) | 414.0(186.0) | 406.5(147.3) | 439.0(364.0) | 0.038* |
| Early recanalization(mTICI≥2b), n (%) | 133(85.3%) | 83(84.7%) | 50(86.2%) | 0.912 |
| NIHSS on discharge as continuous, mean ±S D | 16.91±14.41 | 15.77±14.62 | 18.84±13.97 | 0.198 |
| NIHSS on discharge ≥ 16, n (%) | 63(40.4%) | 34(34.7%) | 29(50.0%) | 0.060 |
| ΔNIHSS as continuous, mean ± SD | -0.08±13.22 | 0.48±13.60 | -1.02±12.61 | 0.496 |
| ΔNIHSS ≥ 4, n (%) | 48(30.8%) | 29(29.6%) | 19(32.8%) | 0.679 |
| **Laboratory data** |  |  |  |  |
| Prothrombin time(s), mean ± SD | 14.84±2.15 | 14.99±2.50 | 14.58±1.35 | 0.283 |
| Fibrinogen(g/L), mean ± SD | 2.84±0.93 | 2.80±0.90 | 2.90±0.90 | 0.557 |
| Activated partial thromboplastin time(s), mean ± SD | 46.18±19.60 | 47.80±20.7 | 43.4±17.3 | 0.210 |
| Thrombin time(s), mean ± SD | 28.74±24.31 | 30.00±24.74 | 26.63±23.69 | 0.460 |
| D-Dimer(mg/L), median (IQR) | 1.6 (2.7) | 1.5(3.2) | 1.8(2.4) | 0.387 |

NIHSS: National Institutes of Health Stroke Scale; ASPECTS: Alberta Stroke Program Early CT score; pc-ASPECTS: posterior circulation Alberta Stroke Program Early CT Score; ΔNIHSS: discharge minus admission NIHSS score; mTICI: modified thrombolysis in cerebral infarction.

*Significant P value.
